# Case of Obstructed Labor from an Extososis, and Adhesion of Vaginal Walls—Operation for Cutting the Stricture—Craniotomy and Delivery—Death on the Fifth Day

**Published:** 1860-02

**Authors:** G. W. Phillips

**Affiliations:** Dixon, Ill.


THE CHICAGO
### MEDICAL JOURNAL.

VOL. III. FEBRUARY, 1860. NO. 2.

### Original Communications.

### ARTICLE I.

### CASE OF OBSTRUCTED LABOR,

FROM AN EXOSTOSIS, AND ADHESION OF VAGINAL WALLS—OPERATION FOR CUTTING THE STRICTURE—CRANIOTOMY AND DELIVERY—DEATH ON THE FIFTH DAY.

REPORTED BY G. W. PHILLIPS, M. D. OF DIXON, ILL.

Before giving the particulars of this case, it will be necessary to give a description of the first labour, with the circumstances that led to the chief complication, (the occlusion of vagina) the facts being furnished me by Dr. N. W. Abbott.

A year and a half ago, Mrs. McG—— had her first labour; the labour was obstructed by the narrowing of the antero-posterior diameter of the superior strait, by a tumour (probably an exostosis) growing from the promontory of the sacrum and last lumbar vertebra—the labour not being completed until the fifth day; the second stage occupying more than forty-eight hours. The head presented in the first position, and as the pains were strong, and exhausting, the attendiug Physicians performed craniotomy, afterwards ergot was given, then forceps used; convulsions occurring, an attempt was made to turn, all however without success. At this stage, the patient was seen by Dr. Abbott, who found her having frequent convulsions and unconscious; the head partly collapsed, was lying in the pelvic cavity, with one foot by its side; returning the foot above the head, he soon delivered with the blunt hook.

The patient was left in a very prostrate condition, and unconscious, her Physicians not expecting her to live but a few hours; she was however, in a few days reported as doing well.

Danger from adhesions of vaginal walls was pointed out to her consequent upon sloughing from long continued pressure of head; and the necessary treatment proposed; the patient however refused to be treated; consequently adhesion of vaginal walls took place.

Within nine months, she again became pregnant. During the early months she was examined by several physicians who were unable to pass a small probe beyond the stricture, there seeming to be complete occlusion of vagina. An operation was advised, but refused.

Such were the complications attending the second labor of Mrs. McG., to which I was called by Dr. Abbott, in consultation, the morning of the 10th of November last. Age of patient, twenty-five; stout, hearty looking Irish woman.

Labor commenced twelve hours previously; pains regular, and gaining force; upon examination, we found, two inches within the vagina, a thin, firm fold or band encircling the entire circumference of the vagina, and into which the end of the finger could barely pass, the edge feeling tense and sharp; the pains growing stronger; the stricture soon dilated so as to admit the finger into the cavity of the pelvis, finding the os largely dilated, and head engaging in superior strait in second position; the liquor amnii having already been evacuated; it was evident that the labor could be successfully terminated only by the dilatation or rupture of the band, by pressure of the advancing head, or by division of the stricture with the knife. After waiting several hours to observe the dilatability of the stricture, or to see if the border would give way, the stricture having dilated so as to admit two fingers breadth, the border becoming very tense, pains heavy, and fearing rupture of vagina above the stricture, we decided to cut the band.

By a careful examination, the stricture was found to be well defined, and formed by adhesion of the mucous membrane only; the thickest and densest part being upon the right side of vagina. Giving the patient chloroform, the band was cut transversely and to the right, the band immediately spreading apart so as to admit three fingers breadth; the pains continued regular and strong, the head slowly advancing and dilating the band, until midnight, when they gradually became weak, so that by next morning they were inefficient to advance the head.

The passage of the head through the superior strait was delayed, not only by the narrowing of the strait, but by the uterus being tilted to the right by the exostosis, and kept there, so that the axis of uterus and strait did not correspond, the force thus exerted by uterus being wasted. Ergot was now given, causing the pains to become stronger, bringing the head to engage in inferior strait; the strait again becoming tense, was again cut, expanding so as to give sufficient room for head to pass; the head advanced, beginning to form the perineal tumor, when symptoms of exhaustion, which had shown themselves for the last six hours, became very evident; pulse quick and growing weaker, tendency to syncope; on being raised in bed, the pains becoming slight, inefficient and exhausting.

We had for several hours suspected that the child was dead, as the fœtal heart could not be heard, and the fluids from the vagina were very fetid; these signs added to the fact that no tumour formed on scalp, and bones of cranium shut fast, so as to close fontanelles made it pretty evident that the child was dead, and had been so some time. As the life of the child was out of the question, it was clear that artificial delivery must be effected speedily; accordingly craniotomy was performed, brain broken down, and head delivered by means of blunt-hook. By traction upon cord placenta soon followed, uterus contracted down well; the child was large and fully developed, the color of cord and membranes was a dirty yellow, the cord was soft and crepitated between the fingers—evidences of putrefaction. The substance of placenta was softened and easily torn, but not decomposed as much as cord; the child showed no marks of decomposition.

The death of the ovum probably took place within a few weeks of labor; what could have produced the death of the ovum is difficult to say; there was no structural degeneration of placenta, cartilaginous or otherwise; no induration from deposit of lymph in substance of placenta, or deposit of lymph upon its surfaces, showing evidence of inflammation; placenta of usual size.

The labor occupied forty-two hours, twelve hours for first stage, and thirty for second.

The patient was ordered stimulants and opium, and left in a favorable condition, except exhaustion, from a prolonged second stage. Forty-eight hours after, when seen by Dr. Abbott, she was still weak, pulse quick, otherwise comfortable; the uterus about the size of one's head, but contracted down; upon applying pressure coagula were expelled. Forty-eight hours from this time, Dr. A. was sent for, as twelve hours previously the patient had had a chill, the cabin being cold and damp, in consequence of there having been a big washing in it on that day. The Dr. found her unconscious, skin cold, pulse thready and weak, pupils dilated, respiration weak, no tenderness or fullness of abdomen; uterus contracted down well; she died twelve hours after. No post-mortem examination.

What was the cause of death? She did not die from inflammation, as a result of an obstructed labor, and the operation for cutting the stricture; nor of exhaustion, from a prolonged second stage, as the symptoms, at no time, previous to the chill approached a collapse. I can find but one probable cause of death, and that is the absorption into the blood of a poison from the putrefying cord and placenta, prostrating the nervous energy so completely, that reaction did not take place after the initiatory chill, which if it had, the phenomena would have been those of puerperal fever.

---

QUINA IN FEBRILE EXACERBATIONS.—The veteran, Dr. Cartwright of New Orleans, in a letter published in the Medical Journal of North Carolina, claims the origination of the use of Quina in large doses in febrile exacerbations. In April, 1826, he recommended this now standard practice, particularly in Pneumonia Biliosa.

